# Prevalence and antimicrobial susceptibility of *Staphylococcus aureus* isolated from dairy goats in Shaanxi, China, with genomic characterization of a multidrug-resistant subset

**DOI:** 10.1016/j.onehlt.2026.101443

**Published:** 2026-05-15

**Authors:** Xindong Bai, Enying Diao, Yuetong Sun, Xin Tian, Bingjun Shi, Yunjie Jiao, Guangming Zhao, Heping Zhao, Mei Yang, Dongyang Ye, Leina Dou, Juan Wang, Zengqi Yang

**Affiliations:** a Department of Preventive Veterinary Medicine, College of Veterinary Medicine, Northwest A&F University, Yangling, China; b Key Laboratory for Prevention and Control of Major Ruminant Diseases, Ministry of Agriculture and Rural Affairs, Yangling, China; c Shaanxi Animal Disease Control and Prevention Center, Xi'an, China

**Keywords:** Dairy goats, *Staphylococcus aureus*, AMR, ST59, ST398, Host spillover risk

## Abstract

*Staphylococcus aureus* (*S. aureus*) is a major etiological agent of mastitis in dairy goats and a potential reservoir of antimicrobial resistance (AMR). In the present study, the prevalence and antimicrobial susceptibility of *S. aureus* isolated from goat milk in Shaanxi Province, China, was investigated and the genomic features of a multidrug-resistant (MDR) subset were characterized. A total of 184 (10.7%) *S. aureus* isolates were recovered from 1720 milk samples collected from 19 dairy farms. Antimicrobial susceptibility testing against 18 agents showed that the isolates exhibited an overall favorable susceptibility profile, with resistance remaining uncommon for most drugs tested. However, nine isolates (4.9%) were classified as MDR, including three (1.6%) methicillin-resistant *S. aureus* (MRSA) isolates. Although MDR was uncommon in the sample, genomic analysis showed that the MDR subset comprised a limited number of high-risk lineages, including MRSA ST59 and methicillin-susceptible *S. aureus* (MSSA) ST398. Comparative phylogenetic analysis indicated that the MRSA ST59 and MSSA ST398 isolates were genetically related to publicly available human and porcine-associated genomes, respectively, consistent with possible host spillover. In addition, the MSSA ST398 and ST522 isolates contained putative host-associated virulence factor variants that warrant further functional validation. Overall, the present study provides region-specific surveillance data on milk-associated *S. aureus* in dairy goats and highlights the importance of continued integrated surveillance of high-risk *S. aureus* lineages within the One Health framework.

## 1 Introduction

Mastitis, primarily caused by bacterial infection, is one of the most economically damaging diseases affecting the global dairy industry [1]. *S. aureus* is a major etiological bacterial agent of mastitis in dairy goats, and can adversely affect milk yield, milk quality, and animal welfare, because of low cure rates and frequent recurrence [2]. Antibiotics are the main therapeutic approach for treating mastitis in dairy goats. However, its irrational or excessive use has accelerated the emergence and spread of bacterial resistance, increasing the risk of treatment failure and potentially facilitating the transmission of resistant strains among animals, the environment, and humans, thereby posing a latent threat to public health [3], [4]. Hence, conducting dynamic epidemiological surveillance and systematic assessment of antimicrobial resistance (AMR) in regional production systems, are of immediate practical importance for guiding targeted antibiotic stewardship, limiting the further development of resistance, and safeguarding both, animal productivity and public health.

Shaanxi Province is a major region for dairy goat production region in China, with a stock of nearly 2.8 million animals, including approximately 1.8 million commercial milk-producing goats. Its fresh goat milk output accounts for more than 60% of the total national consumption, underscoring the province's central role in China's dairy goat industry [5]. However, epidemiological data on *S. aureus* derived from dairy goats in this region remain relatively scarce. Previous studies are limited to individual farms, involving relatively small sample sizes, and lacking systematic analysis of prevalence, AMR profiles, and genomic characteristics. In particular, the molecular epidemiology of multidrug-resistant (MDR) and methicillin-resistant *S. aureus* (MRSA) remain poorly understood [6], [7], [8], [9]. Of particular concern in public health relevance, are two lineages, community-associated MRSA (CA-MRSA) ST59 and livestock-associated ST398 (LA-ST398), that are increasingly being reported in diverse livestock populations in China in recent years, indicating an expanding host range and warranting further genomic investigation [10], [11], [12], [13]. However, their prevalence, host association, and genomic relatedness to lineages from other hosts in the dairy goat population of Shaanxi Province, are unclear. Furthermore, the distribution and variation of virulence genes in *S. aureus* from dairy goats remain poorly characterized.

Against this background, the present study was conducted to investigate the prevalence and antimicrobial susceptibility profile of *S. aureus* isolated from dairy goats in Shaanxi Province, and to further characterize the genomic features of the MDR isolates. Comparative phylogenetic analysis was additionally performed to assess the genomic relatedness of selected high-risk lineages to publicly available genomes from other hosts. The findings provide updated region-specific epidemiological and genomic data for intra-mammary *S. aureus* surveillance, antimicrobial stewardship, and future monitoring of host spillover risk within surveillance under the One Health framework.

## 2 Materials and methods

### 2.1 Sample collection

From 2023 to 2024, a total of 1720 milk samples were collected from 1720 goats across 19 intensive farms in four major dairy goat-producing cities in Shaanxi Province, namely Xi'an (XAA and XAB), Baoji (MXA, MXB, LXA, LXB, LXC, LXD, QYA, QYB and QYC), Xianyang (XYA and XYB), and Weinan (FPA, FPB, FPC, FPD, FPE and FPF). Milk samples were collected from clinically healthy dairy goats, defined as animals without signs of mastitis (e.g., mammary gland swelling, fever, or pain) and with normal milk appearance (e.g., no flocculation, off-odor, or discoloration). Given that *S. aureus* is frequently associated with subclinical mastitis, and reliable diagnostic criteria for subclinical mastitis in goats is lacking, no attempt was made to distinguish healthy carriers from animals with subclinical mastitis. Strict aseptic procedures were followed before sampling. The first 3–5 mL of milk was discarded to minimize contamination from the teat surface, after which 5–10 mL of midstream milk was collected in a sterile centrifuge tube. All samples were transported under refrigeration in an ice box and delivered to the laboratory for processing within 24 h.

### 2.2 Isolation and identification

For the isolation and identification of *S. aureus*, 5 mL of milk from each sample was centrifuged. After the supernatant was discarded, the pellet was re-suspended in 5 mL of 7.5% sodium chloride broth and incubated at 37 °C with shaking at 180 rpm for 18 h. This selective enrichment step was used to favor the growth of staphylococci while suppressing part of the background flora. Subsequently, the enriched culture was streaked onto Baird-Parker agar plates and incubated at 37 °C for 45–48 h. Presumptive *S. aureus* colonies were circular, smooth, convex, and moist, with a diameter of 2–3 mm. They were grayish-black to black with a glossy surface and often had a light-colored margin. An opaque zone (precipitate) surrounded each colony, and was typically bordered by an outer clear zone. Representative colonies were selected for purification and subsequently identified by PCR, which targeted the species-specific *nuc* gene (forward primer: GCTGGCATATGTATGGCAAT; reverse primer: TTCGTAAATGCACTTGCTTCA) [14].

### 2.3 Antimicrobial susceptibility testing

The antimicrobial susceptibility of the isolates to 18 antimicrobial agents was determined using the broth microdilution method by a commercial AST panel for aerobic Gram-positive bacilli (Fosun Diagnostics, Shanghai, China). MIC results were interpreted according to the Clinical and Laboratory Standards Institute (CLSI) guidelines (M100, 32nd edition; VET01-A4). The agents tested were penicillin (PEN), amoxicillin-clavulanic acid (A/C), cefoxitin (CFX), ceftiofur (CEF), oxacillin (OXA), erythromycin (ERY), clindamycin (CLI), tilmicosin (TIL), enrofloxacin (ENR), ofloxacin (OFL), sulfafurazole (SF), sulfamethoxazole-trimethoprim (SXT), vancomycin (VAN), tetracycline (TET), florfenicol (FFC), tiamulin (TIA), gentamicin (GEN), and linezolid (LZD). MDR was defined as acquired non-susceptibility to at least one agent in three or more antimicrobial categories [15]. All procedures were performed strictly according to the manufacturer's instructions. *S. aureus* ATCC 29213 was used as the quality control strain.

### 2.4 Whole-genome sequencing, assembly and annotation

Whole-genome sequencing (WGS) was performed on the nine MDR isolates. Genomic DNA was extracted from pure overnight cultures of the *S. aureus* isolates using the TIANamp Bacteria DNA Kit (Tiangen, Beijing, China) according to the manufacturer's instructions. DNA concentration and purity were assessed using a NanoDrop spectrophotometer and a Qubit fluorometer (Thermo Fisher Scientific, USA). Qualified DNA samples were commercially sequenced by Tsingke Biotechnology Co., Ltd. (Xi'an, China). Paired-end sequencing libraries were prepared following the standard Illumina protocol, and sequenced on an Illumina HiSeq X platform with 2 × 150 bp reads, targeting a minimum average depth of 100× per genome. Raw reads were subjected to quality control, including adapter removal and filtering of low-quality reads, to generate clean data. Genome assembly was performed using SPAdes v3.11.1. Assembly quality was assessed based on genome size, number of contigs, N50, longest contig length, GC content, and completeness. These summary statistics for the nine MDR isolates are provided in Supplementary Table S1. Genome annotation was performed using the RAST online platform tool [16] and Prokka v1.14.6 [17]. WGS data for the nine MDR isolates were deposited into GenBank under BioProject accession number PRJNA1402287.

### 2.5 Comparative genomic and phylogenetic analysis

Publicly available genomes of ST59 and ST398 were retrieved from the NCBI database for comparative analysis. Genomes were selected according to the following criteria: (i) sequence type confirmed as ST59 or ST398; (ii) host source and geographic information available; and (iii) assembly quality sufficient for comparative SNP analysis. Redundant genomes and genomes with incomplete metadata or poor assembly quality were excluded. Metadata for the public genomes included in the present study are provided in Supplementary Tables S2 and S3. Comparative phylogenetic analysis was restricted to ST59 and ST398 because these lineages were of particular public health relevance.

In silico multilocus sequence typing (MLST), spa typing and Staphylococcal cassette chromosome *mec* (SCC*mec*) typing was performed on the assembled genome sequences using MLST 2.0 [18], spaTyper 1.0 [19] and SCC*mec*Finder 1.2 [20], respectively, on the Center for Genomic Epidemiology platform (https://www.genomicepidemiology.org). Resistance genes were identified using ResFinder v4.5.0 [21] and the Comprehensive Antibiotic Resistance Database (CARD) [22]. Virulence genes were annotated based on the Virulence Factors of Pathogenic Bacteria Database (VFDB) [23]. The default parameters were used unless specified otherwise.

Core-genome single nucleotide polymorphism (SNP) analysis was performed using Snippy v4.6.0 (https://github.com/tseemann/snippy). For the ST59 dataset, the *S. aureus* reference genome GCF_000237125.2 was used for SNP calling, whereas the reference genome GCF_024916105.1 was used for the ST398 dataset. Pairwise core-genome SNP distances were extracted from the resulting alignments, and used to describe within-lineage genomic relatedness. Maximum-likelihood phylogenetic trees were constructed using FastTree v2.1.11 [24] based on the core-genome SNP alignments and visualized using iTOL [25].

For analysis of ruminant-associated virulence factors, amino acid sequences of vWbp and leukocidins were aligned and single-gene phylogenetic trees were constructed using MEGA 12 [26]. The SCC*mec att*-site sequences of the goat-derived MSSA ST398 isolates were compared with those of selected porcine-derived ST398 isolates from Qinghai Province to identify SCCmec excision scar sequences.

## 3 Results

### 3.1 Prevalence of *S. aureus* derived from goat milk in Shaanxi Province

**Fig. 1:**
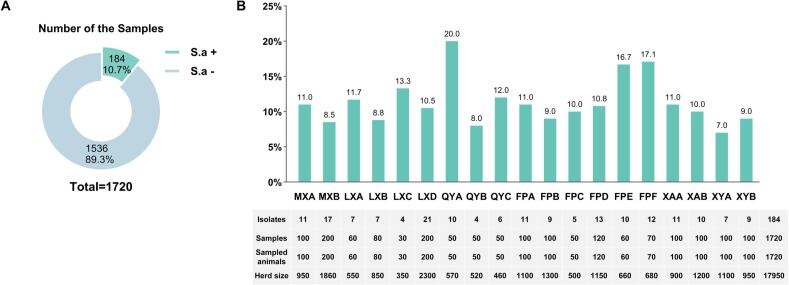
Prevalence of *S. aureus* derived from goat milk in Shaanxi Province. (A) Proportion of *S. aureus*-positive samples among all samples. (B) Isolation rate for each farm together with the number of *S. aureus* isolates, the number of sampled goats, and the total herd size. Individual dairy goat farms are denoted by codes (MXA, MXB, …, XYB).

From 1720 milk samples, bacteriological isolation identified 184 *S. aureus* isolates, corresponding to an overall prevalence of 10.7% (184/1720) (Fig. 1A). At the farm level, prevalence varied markedly, ranging from 7.0% to 20.0%, and representing a greater than twofold difference. Most farms (16/19) had prevalence values between 7.0% and 13.3%, whereas a minority (3/19) showed rates above 15.0% (Fig. 1B).

### 3.2 Antimicrobial resistance profiles of *S. aureus* isolates

**Fig. 2:**
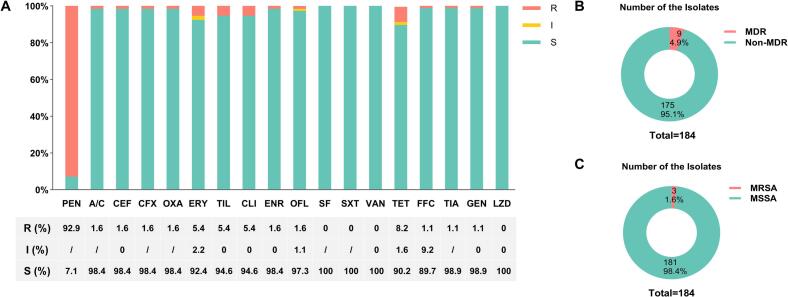
Antimicrobial susceptibility profiles of the *S. aureus* isolates. (A) Distribution of isolates by susceptibility category (Resistant, R; Intermediate, I; Susceptible, S) to 18 antimicrobial agents. (B) Proportion of MDR isolates. (C) Proportion of MRSA isolates. Penicillin (PEN); Amoxicillin-clavulanic acid (A/C); Cefoxitin (CFX); Ceftiofur (CEF); Oxacillin (OXA); Erythromycin (ERY); Clindamycin (CLI); Tilmicosin (TIL); Enrofloxacin (ENR); Ofloxacin (OFL); Sulfafurazole (SF); Sulfamethoxazole-trimethoprim (SXT); Vancomycin (VAN); Tetracycline (TET); Florfenicol (FFC); Tiamulin (TIA); Gentamicin (GEN); and Linezolid (LZD).

To assess the antimicrobial resistance profiles of the 184 isolates, susceptibility to 18 antimicrobial agents was determined. Resistance was most frequently observed to PEN (92.9%), followed by TET (8.2%), whereas no resistance was detected to SXT, SF, VAN, or LZD. Resistance to ERY, CLI, and TIL was each detected in 5.4% of isolates, whereas resistance to each of the remaining nine agents was below 2.0% (Fig. 2A). Overall, nine isolates (4.9%) met the definition of MDR (Fig. 2B), including three MRSA isolates (1.6%) (Fig. 2C).

**Fig. 3:**
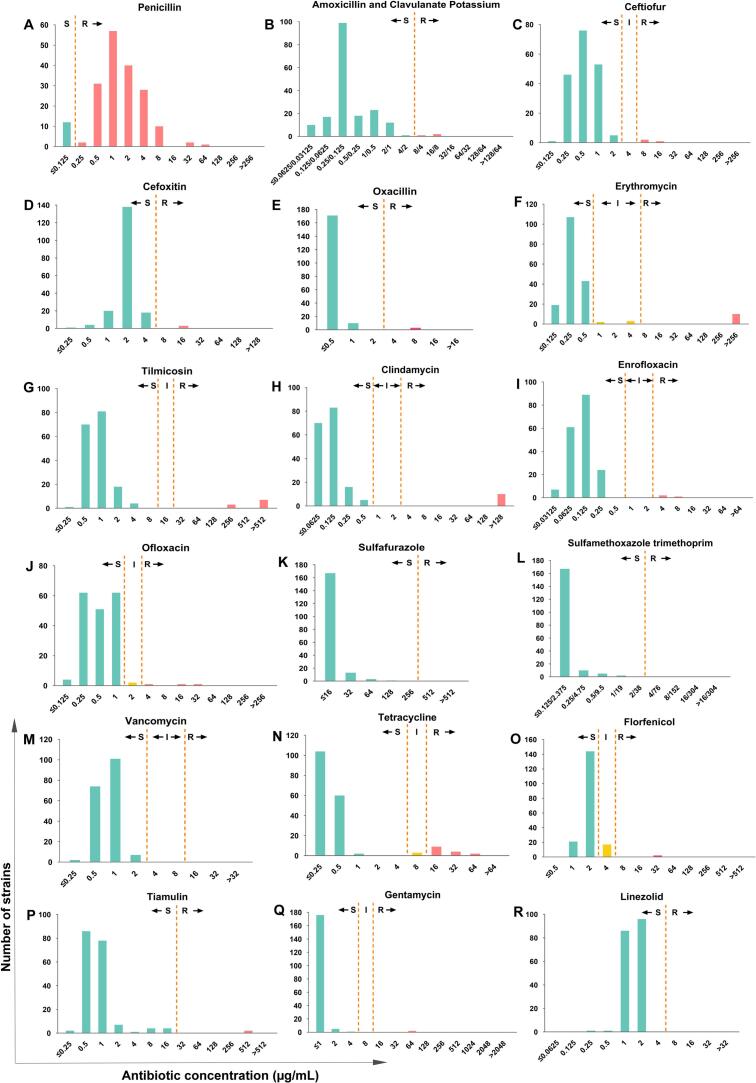
Distribution of MIC values of the 184 *S. aureus* isolates. Resistant (R); Intermediate (I); Susceptible (S).

Although resistance rates were low overall, the MIC distributions for ERY, CLI, TIL, and TIA showed bimodal or right-shifted patterns, indicating the presence of a small subset of highly resistant isolates (Fig. 3F, G, H, and P). Notably, 78.3% of the isolates exhibited an FFC MIC of 2 μg/mL, corresponding to the susceptibility breakpoint (Fig. 3O).

### 3.3 Molecular characteristics of the MDR isolates

As noted above, nine *S. aureus* isolates were classified as MDR strains. To investigate the genetic basis and molecular characteristics of these MDR isolates, WGS and bioinformatics analysis were performed. A detailed summary of molecular typing results and resistance gene profiles is provided in Table 1**.** MLST showed that the nine MDR isolates comprised four ST522, three ST59, and two ST398 strains. All three ST59 isolates were recovered from the same farm, whereas the two ST398 isolates were recovered from another. Notably, isolates within the same MLST type exhibited identical resistance phenotypes and resistance gene profiles. All observed resistance phenotypes were supported by corresponding resistance genes. The high-level resistance to ERY, CLI, and TIL in MDR isolates was attributed to the presence of *erm(C)* in ST522, *erm(B)* in ST59, or the combined presence of *erm(T), lnuB* and *lsaE* in ST398*.* In addition, the present study detected several resistance genes for which corresponding phenotypes were not assessed in the antimicrobial susceptibility testing panel. These included, *aadE* (streptomycin) and *aph(3′)-III* (kanamycin/neomycin) in the ST59 isolates, and *dfrG* (trimethoprim), *aadE* (streptomycin), and *aadD* (tobramycin/kanamycin) in the ST398 isolates. The two ST398 isolates were resistant to ten tested agents representing eight antimicrobial classes, and carried the largest number of resistance genes (*n* = 12), indicating the broadest resistance spectrum observed. The three ST59 isolates were resistant to nine tested agents representing four antimicrobial classes and carried six resistance genes. All three were characterized as MRSA carrying SCC*mec* type IVa, a small, community-associated, and highly mobile genetic cassette. The four ST522 isolates were resistant to four tested agents representing three antimicrobial classes and carried two resistance genes.

**Table 1:** Molecular characteristics of the MDR isolates.

Isolates	MLST	SCC*mec*	PEN	A/C	CEF	CFX	OXA	ERY	TIL	CLI	ENR	OFL	SF	SXT	VAN	TET	FFC	TIA	GEN	LZD	Resistance gene profiles
QYC CH4	ST522		2	0.25/0.12	0.25	2	≤0.5	>256	>128	>512	0.12	0.5	≤16	≤0.12/2.4	1	≤0.25	2	1	≤1	1	*blaZ-erm(C)*
QYC CH5	ST522		2	0.25/0.12	0.25	2	≤0.5	>256	>128	>512	0.12	0.5	≤16	≤0.12/2.4	1	≤0.25	2	1	≤1	1	*blaZ-erm(C)*
XAB CH9	ST522		2	0.25/0.12	0.25	2	≤0.5	>256	>128	>512	0.12	0.5	≤16	≤0.12/2.4	1	≤0.25	2	1	≤1	1	*blaZ-erm(C)*
FPF CH5	ST522		2	0.25/0.12	0.25	2	≤0.5	>256	>128	>512	0.12	0.5	≤16	≤0.12/2.4	1	≤0.25	4	1	≤1	1	*blaZ-erm(C)*
XYA CH1	ST59	IVa	32	8/4	8	16	8	>256	>128	256	0.25	1	≤16	≤0.12/2.4	1	16	2	1	≤1	2	*blaZ-mecA-erm(B)-tet(K)-aadE-aph(3′)-III*
XYA CH2	ST59	IVa	64	16/8	8	16	8	>256	>128	256	0.25	1	≤16	≤0.12/2.4	1	16	2	1	≤1	2	*blaZ-mecA-erm(B)-tet(K)-aadE-aph(3′)-III*
XYA CH3	ST59	IVa	32	16/8	16	16	8	>256	>128	256	0.25	1	≤16	≤0.12/2.4	1	16	2	1	≤1	2	*blaZ-mecA-erm(B)-tet(K)-aadE-aph(3′)-III*
XAA CH10	ST398		16	0.25/0.12	1	2	≤0.5	>256	>128	>512	8	32	≤16	0.25/4.8	0.5	64	32	512	64	1	*blaZ-erm(T)-lnuB-lsaE-gyrA(S84L)-dfrG-tet(M)-tet(L)-* *fexA-aadE-aadD-aac(6′)-aph(2″)*
XAA CH11	ST398		4	0.25/0.12	1	2	≤0.5	>256	>128	>512	4	16	≤16	≤0.12/2.4	0.5	64	32	512	64	1	*blaZ-erm(T)-lnuB-lsaE-gyrA(S84L)-dfrG-tet(M)-tet(L)-* *fexA-aadE-aadD-aac(6′)-aph(2″)*

Note: Shading indicates MIC values above the resistance breakpoint. Penicillin (PEN); amoxicillin-clavulanic acid (A/C); cefoxitin (CFX); ceftiofur (CEF); oxacillin (OXA); erythromycin (ERY); clindamycin (CLI); tilmicosin (TIL); enrofloxacin (ENR); ofloxacin (OFL); sulfafurazole (SF); sulfamethoxazole-trimethoprim (SXT); vancomycin (VAN); tetracycline (TET); florfenicol (FFC); tiamulin (TIA); gentamicin (GEN); and linezolid (LZD).

### 3.4 Phylogenetic relationships and comparative genomic characteristics of MRSA ST59 and MSSA ST398 isolates

**Fig. 4:**
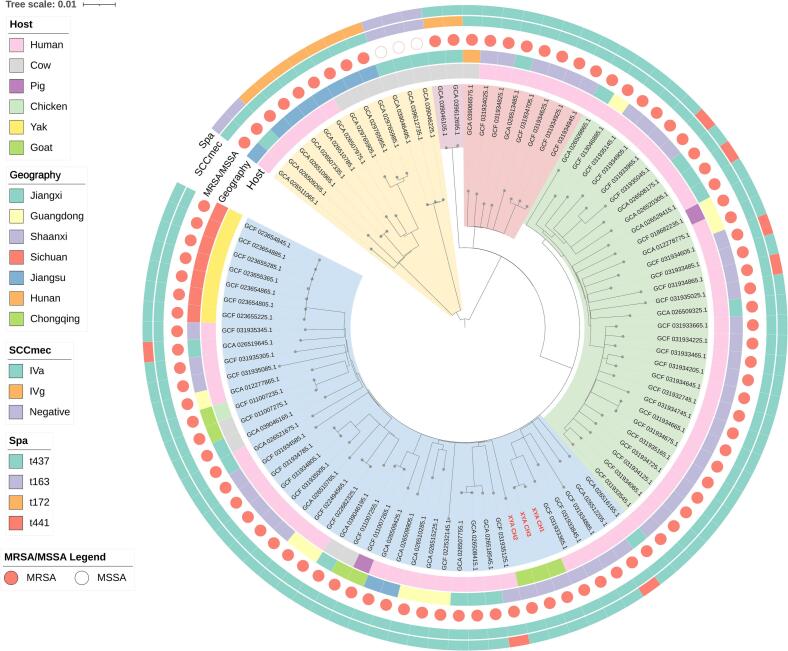
Phylogenetic relationship of *S. aureus* ST59 isolates from dairy goat milk in Shaanxi Province. The ST59 isolates identified in the present study are marked in red font. Host source, geographic origin, SCC*mec* type, spa type and presence of *mecA* are indicated in the outer rings. (For interpretation of the references to colour in this figure legend, the reader is referred to the web version of this article.)

Comparative phylogenetic analysis based on core-genome SNPs was performed for the goat-derived MRSA ST59 and MSSA ST398 isolates, using publicly available genomes from different hosts. It showed that the three goat-derived MRSA ST59 isolates were closely related to selected human-derived ST59 genomes (Fig. 4). Among the publicly available ST59 genomes, the nearest non-goat genome to XYA CH1 and XYA CH3 was the human-derived GCF_031933365.1, differing by 92 and 88 core SNPs, respectively, whereas XYA CH2 was most closely related to the human-derived genome GCA_026515225.1, differing by 138 core SNPs. Pairwise core-genome SNP analysis showed that the three goat-derived MRSA ST59 isolates were not equally related. XYA CH1 and XYA CH3 differed by only 6 core SNPs, whereas XYA CH2 differed from XYA CH1 and XYA CH3 by 180 and 176 core SNPs, respectively, indicating that CH1 and CH3 were more closely related to each other than to CH2, while CH2 represented a more divergent ST59 genotype within the same lineage. Moreover, all the three MRSA ST59 isolates carried the immune evasion cluster (IEC) associated with human adaptation, including *sak*, *chp*, and *scn* (Supplementary Fig. S1).

**Fig. 5:**
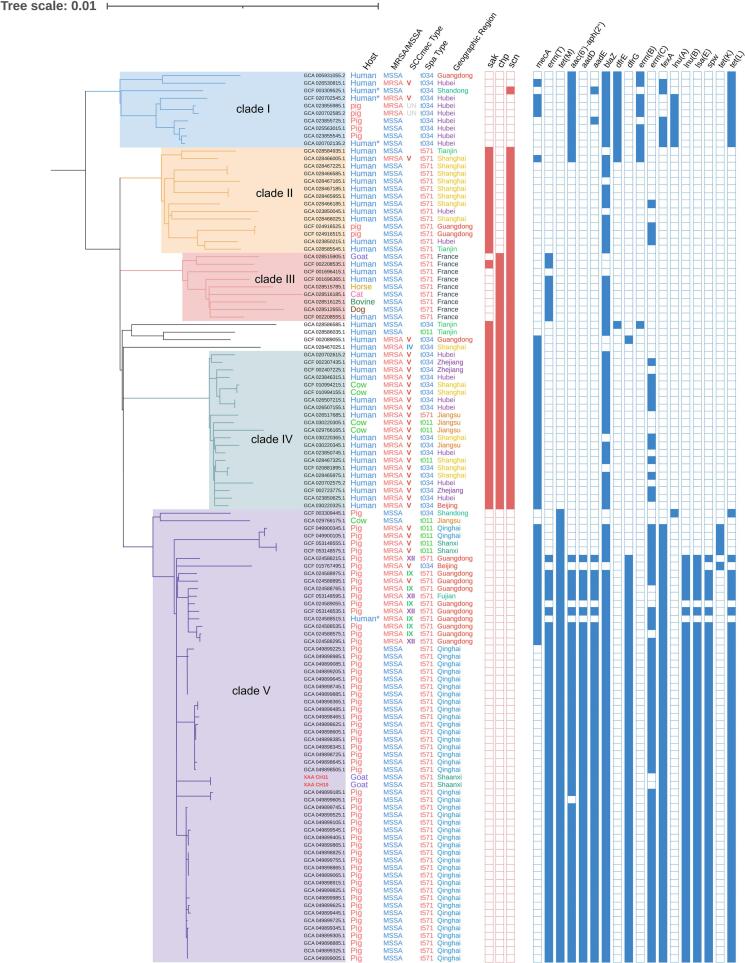
Phylogenetic relationship of *S. aureus* ST398 isolates from dairy goat milk in Shaanxi Province. The ST398 isolates identified in the present study are marked in red font. Human* refers to individuals with a history of contact with pigs. UN, unknown. (For interpretation of the references to colour in this figure legend, the reader is referred to the web version of this article.)

**Fig. 6:**
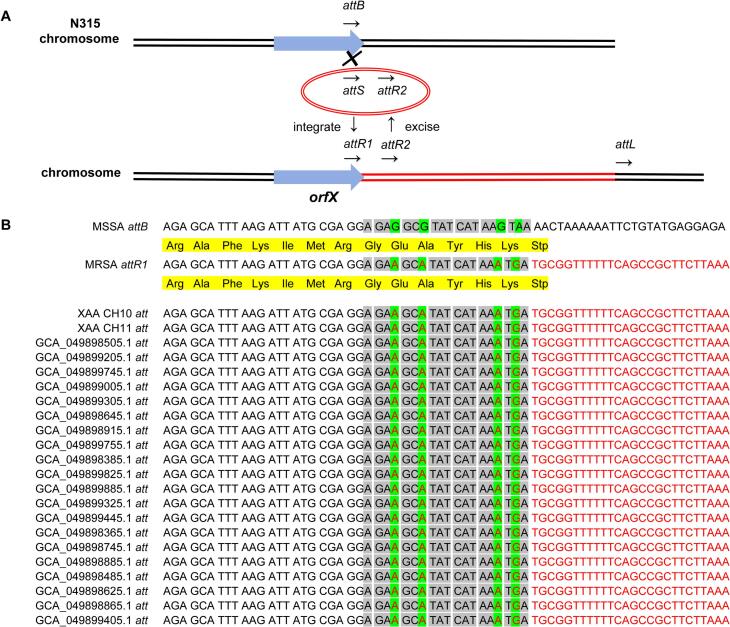
DNA sequence characteristics of *att* sites. (A) Schematic diagram of SCC*mec* integration and excision. DNA sequences within *att* sites are shown in black if they originate solely from the chromosomal *att* site (*attB*) and in red if they derive from the circular incoming SCC*mec* element [28]. (B) DNA sequences of the *att* sites in XAA CH10 and XAA CH11 from the present study, and in selected isolates from Qinghai Province belonging to clade V in Fig. 5. (For interpretation of the references to colour in this figure legend, the reader is referred to the web version of this article.)

The ST398 genomes formed several clades in the phylogenetic tree, with differences in host source observed among clades. The two goat-derived MSSA ST398 isolates located within clade V, together with porcine-derived MSSA ST398 isolates from Qinghai Province, showed highly similar AMR gene profiles (Fig. 5). Pairwise core-genome SNP analysis showed that XAA CH10 and XAA CH11 differed by 0 SNPs. Both isolates were most closely related to a small group of porcine-derived MSSA ST398 genomes from Qinghai Province, with a minimum distance of 41 core SNPs to the nearest genomes (GCA_049899105.1, GCA_049899525.1, GCA_049898915.1, and GCA_049899825.1). This data indicates close genetic relatedness between the goat-derived ST398 isolates and selected porcine-derived genomes. Since LA-ST398 is frequently reported as MRSA [27], the present study further analyzed the SCC*mec att*-site sequences of the two goat-derived together with those of porcine-derived MSSA ST398 isolates from Qinghai Province. A scar sequence originating from the SCC*mec* element (*attS*) was identified at the *att* sites of these isolates, although a few Qinghai isolates lacked a complete *orfX* gene (Fig. 6B).

### 3.5 Analysis of ruminant-adapted virulence factors of MDR isolates

**Fig. 7:**
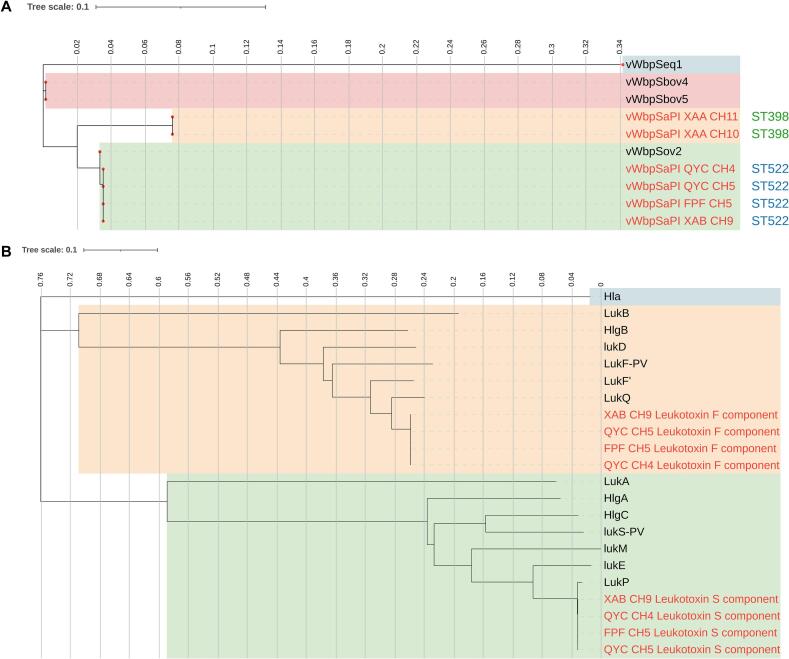
Phylogenetic trees based on amino acid sequences. (A) Phylogenetic analysis of SaPI-carried vWbp. (B) Phylogenetic analysis of leukocidins, with alpha-hemolysin used as an outgroup. The sequences obtained in the present study are highlighted in red. (For interpretation of the references to colour in this figure legend, the reader is referred to the web version of this article.)

To assess the ruminant host adaptability and pathogenic potential of these MDR strains, screening was performed for the host-associated virulence genes that are typically carried by mobile genetic elements (MGEs), such as bacteriophages and pathogenicity islands. The results showed that the MSSA ST398 isolates contained a novel vWbp variant encoded by a SaPI, with 92.4% amino acid identity to vWbp^Sov2^ encoded by SaPIov2 and 91.8% identity to vWbp^Sbov4^ or vWbp^Sbov5^ encoded by SaPIbov4 or SaPIbov5. All four ST522 isolates possessed vWbp^Sov2^, showing 99.9% amino acid identity to the reference vWbp^Sov2^ sequence (Fig. 7A). In addition, all four ST522 isolates carried a LukPQ-like leukocidin encoded by a prophage, which shared 99.9% and 93.3% amino acid identity with LukP and LukQ, respectively (Fig. 7B).

## 4 Discussion

The present study provides region-specific surveillance data on the prevalence and antimicrobial susceptibility of milk-associated *S. aureus* in dairy goats in Shaanxi Province, a major dairy goat production region in China. Although MDR was uncommon overall, the MDR subset contained lineages of public health relevance, including MRSA ST59 and MSSA ST398. Comparative genomic analysis further showed that these goat-derived isolates were genetically related to publicly available human- and porcine-associated genomes, respectively, supporting the value of continued targeted surveillance of high-risk lineages within a broader regional monitoring framework.

Shaanxi Province accounts for more than 60% of China's fresh goat milk output, yet region-wide data on *S. aureus* from dairy goats remain limited [6], [7], [8], [9]. In the present study, the overall isolation rate was 10.7%, slightly lower than the previously reported prevalence of subclinical mastitis-associated *S. aureus* in dairy goats from Shaanxi Province [6]. Since the milk samples were collected from clinically healthy goats, the observed isolation rate is more appropriately interpreted as reflecting *S. aureus* carriage and/or subclinical intramammary infection in apparently healthy herds. Notably, the isolation rate varied from 7.0% to 20.0% across farms, suggesting that the herd-level factors; such as teat disinfection, milking hygiene, and general herd health management; may influence the occurrence of *S. aureus* in this production system.

Phenotypically, the isolate collection showed a relatively favorable antimicrobial susceptibility profile, apart from the very high resistance rate to PEN. This pattern is consistent with the widespread persistence of β-lactamase-mediated PEN resistance in ruminant-associated *S. aureus* [9], [29], [30]. By contrast, resistance to most other tested agents remained low, and MDR was identified in only 4.9% of isolates, suggesting that broad MDR is not yet widespread in this population. Nevertheless, the FFC MIC distribution warrants continued observation: although all isolates remained susceptible, 78.3% exhibited an MIC of 2 μg/mL, corresponding to the susceptibility breakpoint. The clinical significance of this pattern remains uncertain, but it merits continued monitoring in future longitudinal surveillance [31].

The detection of MRSA ST59 and MSSA ST398 among the MDR isolates from dairy goats is noteworthy. To the best of the authors' knowledge, this is the first report of these two lineages in dairy goats in Shaanxi Province. Importantly, these high-risk MDR lineages showed evidence of local occurrence at the farm level: the three ST59 isolates originated from the same farm and the two ST398 isolates from another, with within-lineage distances of 6 and 0 core SNPs, respectively. Taken into account together, these findings are consistent with localized occurrence or possible on-farm dissemination of selected MDR clones.

ST59 is a major CA-MRSA lineage in China [32], [33]. In the present study, the goat-derived ST59 isolates were genetically related to human-derived ST59 genomes from China, and all carried the IEC genes *sak*, *chp*, and *scn*, which are commonly found in human-associated *S. aureus* lineages. Pairwise SNP analysis further showed that XYA CH1 and XYA CH3 were closely related to each other (6 core SNPs), whereas XYA CH2 was more distant from both isolates (176–180 core SNPs), suggesting the presence of at least two genetically differentiated ST59 sublineages among the goat-derived isolates. The nearest selected human-derived genomes differed by 88–138 core SNPs from the goat isolates, indicating a genetic relatedness but not sufficient evidence to infer recent direct transmission or transmission directionality. Furthermore, MRSA ST59 has increasingly been detected in multiple livestock species in China, including dairy cows, pigs, retail pork and yaks [10], [12], [13], [34], [35], [36], [37]. The occurrence of ST59 in multiple livestock species is consistent with an expanded ecological distribution and highlights its relevance within the One Health framework.

ST398 is the predominant LA-MRSA lineage in Europe, whereas ST9 is more commonly reported in China [38], [39]. However, ST398 has increasingly been reported in pig production systems in some regions of China [11], [36], [40], [41], [42]. LA-ST398 is typically characterized by the acquisition of *tet(M)* and the absence of IEC genes, whereas human-associated MSSA ST398 more often carries *erm(T)* together with IEC genes [43], [44]. In the present study, the two goat-derived MSSA ST398 isolates carried both *erm(T)* and *tet(M)* but lacked IEC genes. Phylogenetically, they clustered within a livestock-associated clade together with porcine-derived ST398 isolates from Qinghai Province, and were separated from human-derived lineages. Pairwise SNP analysis showed that XAA CH10 and XAA CH11 differed by 0 core-genome SNPs, and that their closest porcine-derived ST398 genomes differed by 41 core SNPs, supporting close phylogenetic relatedness to previously reported porcine-derived ST398 isolates, while still falling short of proving a recent transmission event. Accordingly, the present data supports the possibility that the goat-derived MSSA ST398 isolates belong to an LA-ST398 background, and show close phylogenetic relatedness to porcine-associated genomes, but further epidemiological sampling and denser genomic comparisons are required to clarify transmission routes and directionality. In addition, the two goat-derived MSSA ST398 isolates shared the characteristic SCC*mec* excision scar sequence with MSSA ST398 isolates from pigs in Qinghai Province, which revealed that these MSSA strains are likely descendants of an MRSA ancestor. In the Qinghai porcine-derived ST398 collection, MSSA ST398 accounted for a higher proportion than MRSA ST398 (60.3% vs. 38.2%) [11], raising the possibility that SCC*mec* loss followed by clonal expansion may contribute to niche adaptation in certain LA-ST398 backgrounds. Previous studies have similarly suggested that SCC*mec* loss may confer a survival advantage to *S. aureus* under adverse conditions [45], [46], [47], [48], [49]. Given the reported host range and MDR potential of the ST398 lineage, continued integrated surveillance remains important within a One Health framework [50], [51], [52].

The identification of novel virulence variants in the MDR isolates suggests ongoing genomic diversification within these lineages. SaPI-encoded vWbps may represent an important mechanism of ruminant adaptation in *S. aureus* pathogenicity [53]. In the present study, all four ST522 isolates, representing a classical caprine-associated lineage, possessed the vWbp^Sov2^, whereas the two MSSA ST398 isolates each contained a novel SaPI-encoded vWbp variant. This may indicate host-associated adaptation, although functional studies are needed to determine the biological relevance of these variants [54]. In addition, all four ST522 isolates carried a LukPQ-like leukocidin, whose contribution to immune evasion or disease severity remains to be experimentally validated [55], [56].

The present study has some limitations. First, the samples were collected from clinically healthy goats, which may have led to underestimation of the prevalence and diversity of *S. aureus* associated with clinical mastitis. Second, WGS was performed only on the nine MDR isolates. Therefore, the genomic analysis presented describes the characteristics of the MDR subset, and do not capture the broader genomic diversity of non-MDR isolates in the study population. Third, the novel virulence factors were inferred from genomic data but were not validated experimentally, such as cytotoxicity assays for the LukPQ-like leukocidin, or ruminant plasma coagulase assays for the novel vWbp variant.

In conclusion, *S. aureus* isolated from dairy goat milk in Shaanxi Province showed a generally favorable antimicrobial susceptibility profile, apart from the high prevalence of PEN resistance. Although MDR was uncommon, the MDR subset contained lineages of public health relevance, including MRSA ST59 and MSSA ST398, and showed localized occurrence within individual farms together with close phylogenetic relatedness to genomes from other hosts. These findings support susceptibility-guided antimicrobial use and continued targeted surveillance of high-risk lineages within a One Health framework.

## CRediT authorship contribution statement

**Xindong Bai:** Writing – original draft, Methodology, Investigation. **Enying Diao:** Methodology. **Yuetong Sun:** Investigation. **Xin Tian:** Methodology. **Bingjun Shi:** Investigation. **Yunjie Jiao:** Investigation. **Guangming Zhao:** Investigation. **Heping Zhao:** Investigation. **Mei Yang:** Investigation. **Dongyang Ye:** Methodology. **Leina Dou:** Writing – review & editing. **Juan Wang:** Writing – review & editing, Methodology, Investigation. **Zengqi Yang:** Supervision, Project administration, Funding acquisition.

## Ethics approval

Milk sampling was performed as part of routine farm management and was non-invasive. All procedures were conducted with the consent of the farm owners and in accordance with institutional guidelines for animal welfare.

## Declaration of generative AI and AI-assisted technologies in the writing process

During the preparation of this work the authors used ChatGPT (OpenAI) to improve readability and language. After using this tool/service, the authors reviewed and edited the content as needed and take full responsibility for the content of the published article.

## Funding statement

This work was supported by the Provincial Modern Agricultural Industry Technology System Construction Project (Grant No. K3021525001) and the Breeding of Dairy Goat Varieties and Comprehensive Application of Supporting Technologies Project (Grant No. K3030225238).

## Declaration of competing interest

The authors declare that they have no known competing financial interests or personal relationships that could have appeared to influence the work reported in this paper.

## Data Availability

The WGS data generated in this study have been deposited in GenBank under BioProject accession number PRJNA1402287. Additional data supporting the findings of this study are available from the corresponding authors upon reasonable request.
