# Supplementary material for: Prevalence and antimicrobial susceptibility of Staphylococcus aureus isolated from dairy goats in Shaanxi, China, with genomic characterization of a multidrug-resistant subset
Source: One Health. 2026 May 15;22:101443. doi: 10.1016/j.onehlt.2026.101443 (PMC13197723; doi:10.1016/j.onehlt.2026.101443)
Supplement: Supplementary file 1 — Supplementary material [file mmc1.docx]

**Supplementary materials**


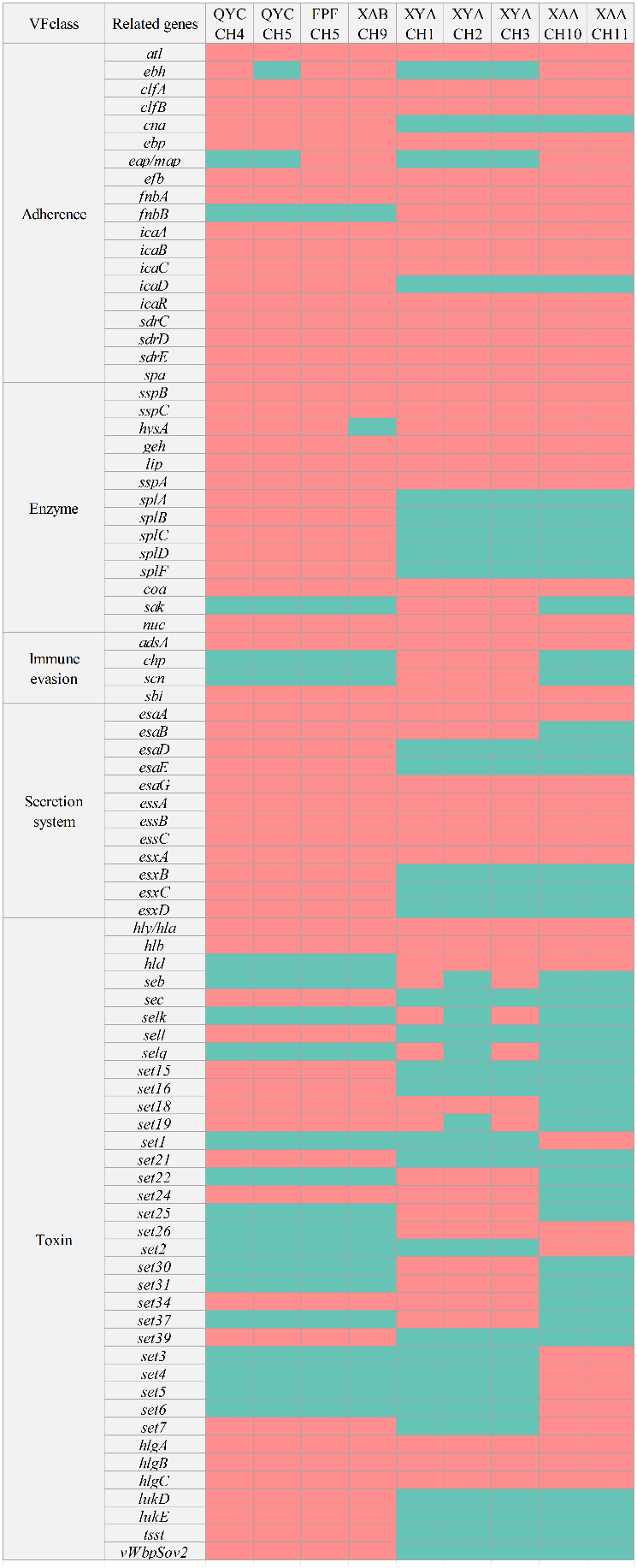


**Supplementary Figure S1.** Toxin gene profiles of the MDR isolates. Shading indicates gene presence (red) or absence (green).

**Supplementary Table S1.** Assembly statistics of the nine MDR isolates

| Isolate | Genome size (bp) | Number of contigs | N50 (bp) | Longest contig (bp) | GC content (%) | Completeness (%) | Contamination (%) |
| --- | --- | --- | --- | --- | --- | --- | --- |
| FPF CH5 | 2,807,826 | 41 | 238,611 | 608,796 | 32.78 | 98.13 | 0.27 |
| QYC CH4 | 2,831,294 | 46 | 238,710 | 608,706 | 32.73 | 98.13 | 0.24 |
| QYC CH5 | 2,831,295 | 46 | 238,710 | 608,706 | 32.73 | 98.13 | 0.24 |
| XAA CH10 | 2,752,573 | 49 | 149,668 | 422,379 | 32.78 | 97.75 | 0.26 |
| XAA CH11 | 2,752,116 | 47 | 149,668 | 422,379 | 32.79 | 97.75 | 0.26 |
| XAB CH9 | 2,805,885 | 50 | 238,680 | 608,796 | 32.79 | 98.13 | 0.27 |
| XYA CH1 | 2,763,224 | 59 | 150,324 | 452,430 | 32.73 | 99.11 | 0.39 |
| XYA CH2 | 2,742,727 | 62 | 133,037 | 319,344 | 32.74 | 98.92 | 0.41 |
| XYA CH3 | 2,760,827 | 64 | 150,326 | 452,443 | 32.73 | 99.11 | 0.39 |

**Supplementary Table S2.** Information of 92 ST59 strains from NCBI database

| Accession number | Host | Province | Spa type | MRSA/MSSA | SCCmec type |
| --- | --- | --- | --- | --- | --- |
| GCA_039046105.1 | Cow | Jiangxi | t437 | MRSA | IVg |
| GCA_039046165.1 | Cow | Jiangxi | t437 | MRSA | IVa |
| GCA_039046195.1 | Cow | Jiangxi | t437 | MRSA | IVa |
| GCA_039046225.1 | Cow | Jiangxi | t163 | MSSA | - |
| GCA_039046495.1 | Cow | Jiangxi | t163 | MSSA | - |
| GCA_039066575.1 | Cow | Hunan | t437 | MRSA | IVa |
| GCA_039612695.1 | Cow | Jiangxi | t437 | MRSA | IVg |
| GCA_039612735.1 | Cow | Jiangxi | t163 | MSSA | - |
| GCF_011007275.1 | Cow | Chongqing | t437 | MRSA | IVa |
| GCF_011007255.1 | Cow | Chongqing | t437 | MRSA | IVa |
| GCF_011007265.1 | Pig | Chongqing | t437 | MRSA | IVa |
| GCF_011007235.1 | Chicken | Chongqing | t437 | MRSA | IVa |
| GCF_018682235.1 | Pig | Guangdong | t437 | MRSA | IVa |
| GCF_023654805.1 | Yak | Sichuan | t437 | MRSA | IVa |
| GCF_023654845.1 | Yak | Sichuan | t437 | MRSA | IVa |
| GCF_023654865.1 | Yak | Sichuan | t437 | MRSA | IVa |
| GCF_023654885.1 | Yak | Sichuan | t437 | MRSA | IVa |
| GCF_023655225.1 | Yak | Sichuan | t437 | MRSA | IVa |
| GCF_023655285.1 | Yak | Sichuan | t437 | MRSA | IVa |
| GCF_023655365.1 | Yak | Sichuan | t437 | MRSA | IVa |
| GCA_029765985.1 | Cow | Jiangsu | t172 | MRSA | IVa |
| GCA_029765955.1 | Cow | Jiangsu | t172 | MRSA | IVa |
| GCA_029765905.1 | Cow | Jiangsu | t172 | MRSA | IVa |
| GCF_022494565.1 | Human | Guangdong | t437 | MRSA | IVa |
| GCF_022532145.1 | Human | Guangdong | t437 | MRSA | IVa |
| GCF_022682325.1 | Human | Guangdong | t437 | MRSA | IVa |
| GCF_013046885.1 | Human | Guangdong | t437 | MRSA | IVa |
| GCA_012277865.1 | Human | Guangdong | t437 | MRSA | IVa |
| GCA_012278775.1 | Human | Guangdong | t437 | MRSA | IVa |
| GCA_026512205.1 | Human | Shaanxi | t441 | MRSA | IVa |
| GCA_026510765.1 | Human | Shaanxi | t437 | MRSA | IVa |
| GCA_026515225.1 | Human | Guangdong | t437 | MRSA | IVa |
| GCA_026510285.1 | Human | Guangdong | t437 | MRSA | IVa |
| GCA_026511065.1 | Human | Jiangsu | t163 | MRSA | IVa |
| GCA_026507335.1 | Human | Jiangsu | t172 | MRSA | IVa |
| GCA_026510965.1 | Human | Jiangsu | t172 | MRSA | IVa |
| GCA_026509905.1 | Human | Jiangsu | t437 | MRSA | IVa |
| GCA_026507975.1 | Human | Jiangsu | t172 | MRSA | IVa |
| GCA_026509425.1 | Human | Jiangsu | t437 | MRSA | IVa |
| GCA_026510785.1 | Human | Jiangsu | t172 | MRSA | IVa |
| GCA_026521675.1 | Human | Jiangxi | t437 | MRSA | IVa |
| GCA_026513485.1 | Human | Jiangxi | t437 | MRSA | IVa |
| GCA_026516165.1 | Human | Jiangxi | t437 | MRSA | IVa |
| GCA_026518545.1 | Human | Jiangxi | t437 | MRSA | IVa |
| GCA_026519645.1 | Human | Jiangxi | t441 | MRSA | IVa |
| GCA_026508415.1 | Human | Jiangxi | t437 | MRSA | IVa |
| GCA_026507755.1 | Human | Jiangxi | t437 | MRSA | IVa |
| GCA_026509265.1 | Human | Jiangxi | t163 | MRSA | IVa |
| GCA_026509325.1 | Human | Jiangxi | t437 | MRSA | IVa |
| GCA_026508175.1 | Human | Jiangxi | t437 | MRSA | IVa |
| GCA_026520305.1 | Human | Jiangxi | t441 | MRSA | IVa |
| GCA_026509865.1 | Human | Jiangxi | t437 | MRSA | IVa |
| GCA_026529415.1 | Human | Jiangxi | t437 | MRSA | IVa |
| GCF_031934225.1 | Human | Shaanxi | t437 | MRSA | IVa |
| GCF_031933845.1 | Human | Shaanxi | t437 | MRSA | IVa |
| GCF_031933365.1 | Human | Shaanxi | t437 | MRSA | IVa |
| GCF_031933965.1 | Human | Shaanxi | t437 | MRSA | IVa |
| GCF_031933665.1 | Human | Shaanxi | t437 | MRSA | IVa |
| GCF_031933545.1 | Human | Shaanxi | t437 | MRSA | IVa |
| GCF_031933485.1 | Human | Shaanxi | t437 | MRSA | IVa |
| GCF_031934065.1 | Human | Shaanxi | t437 | MRSA | IVa |
| GCF_031934125.1 | Human | Shaanxi | t437 | MRSA | IVa |
| GCF_031934025.1 | Human | Shaanxi | t437 | MRSA | IVa |
| GCF_031933465.1 | Human | Shaanxi | t437 | MRSA | IVa |
| GCF_031935345.1 | Human | Shaanxi | t437 | MRSA | IVa |
| GCF_031935025.1 | Human | Shaanxi | t437 | MRSA | IVa |
| GCF_031935305.1 | Human | Shaanxi | t437 | MRSA | IVa |
| GCF_031935165.1 | Human | Shaanxi | t437 | MRSA | IVa |
| GCF_031935145.1 | Human | Shaanxi | t437 | MRSA | IVa |
| GCF_031935085.1 | Human | Shaanxi | t437 | MRSA | IVa |
| GCF_031934925.1 | Human | Shaanxi | t437 | MRSA | IVa |
| GCF_031935005.1 | Human | Shaanxi | t437 | MRSA | IVa |
| GCF_031934885.1 | Human | Shaanxi | t437 | MRSA | IVa |
| GCF_031932745.1 | Human | Shaanxi | t437 | MRSA | IVa |
| GCF_031934945.1 | Human | Shaanxi | t437 | MRSA | IVa |
| GCF_031935045.1 | Human | Shaanxi | t441 | MRSA | IVa |
| GCF_031935125.1 | Human | Shaanxi | t441 | MRSA | IVa |
| GCF_031934745.1 | Human | Shaanxi | t437 | MRSA | IVa |
| GCF_031934585.1 | Human | Shaanxi | t437 | MRSA | IVa |
| GCF_031934905.1 | Human | Shaanxi | t437 | MRSA | IVa |
| GCF_031934805.1 | Human | Shaanxi | t437 | MRSA | IVa |
| GCF_031934785.1 | Human | Shaanxi | t437 | MRSA | IVa |
| GCF_031934825.1 | Human | Shaanxi | t437 | MRSA | IVa |
| GCF_031934725.1 | Human | Shaanxi | t437 | MRSA | IVa |
| GCF_031934705.1 | Human | Shaanxi | t437 | MRSA | IVa |
| GCF_031934675.1 | Human | Shaanxi | t437 | MRSA | IVa |
| GCF_031934665.1 | Human | Shaanxi | t437 | MRSA | IVa |
| GCF_031934645.1 | Human | Shaanxi | t437 | MRSA | IVa |
| GCF_031934625.1 | Human | Shaanxi | t437 | MRSA | IVa |
| GCF_031934565.1 | Human | Shaanxi | t437 | MRSA | IVa |
| GCF_031934865.1 | Human | Shaanxi | t441 | MRSA | IVa |
| GCF_031934605.1 | Human | Shaanxi | t441 | MRSA | IVa |

**Supplementary Table S3.** Information of 128 ST398 strains from NCBI database

| Accession number | Host | MRSA/MSSA | SCCmec | *spa* type | region |
| --- | --- | --- | --- | --- | --- |
| GCA_049899985.1 | Pig | MSSA |  | t571 | China:Qinghai |
| GCA_049899865.1 | Pig | MSSA |  | t571 | China:Qinghai |
| GCA_049899885.1 | Pig | MSSA |  | t571 | China:Qinghai |
| GCA_049899825.1 | Pig | MSSA |  | t571 | China:Qinghai |
| GCA_049899755.1 | Pig | MSSA |  | t571 | China:Qinghai |
| GCA_049899745.1 | Pig | MSSA |  | t571 | China:Qinghai |
| GCA_049899725.1 | Pig | MSSA |  | t571 | China:Qinghai |
| GCA_049899625.1 | Pig | MSSA |  | t571 | China:Qinghai |
| GCA_049899645.1 | Pig | MSSA |  | t571 | China:Qinghai |
| GCA_049899605.1 | Pig | MSSA |  | t571 | China:Qinghai |
| GCA_049899545.1 | Pig | MSSA |  | t571 | China:Qinghai |
| GCA_049899525.1 | Pig | MSSA |  | t571 | China:Qinghai |
| GCA_049899445.1 | Pig | MSSA |  | t571 | China:Qinghai |
| GCA_049899405.1 | Pig | MSSA |  | t571 | China:Qinghai |
| GCA_049899345.1 | Pig | MSSA |  | t571 | China:Qinghai |
| GCA_049899325.1 | Pig | MSSA |  | t571 | China:Qinghai |
| GCA_049899305.1 | Pig | MSSA |  | t571 | China:Qinghai |
| GCA_049899225.1 | Pig | MSSA |  | t571 | China:Qinghai |
| GCA_049899205.1 | Pig | MSSA |  | t571 | China:Qinghai |
| GCA_049899185.1 | Pig | MSSA |  | t571 | China:Qinghai |
| GCA_049899105.1 | Pig | MSSA |  | t571 | China:Qinghai |
| GCA_049899085.1 | Pig | MSSA |  | t571 | China:Qinghai |
| GCA_049899065.1 | Pig | MSSA |  | t571 | China:Qinghai |
| GCA_049898985.1 | Pig | MSSA |  | t571 | China:Qinghai |
| GCA_049899005.1 | Pig | MSSA |  | t571 | China:Qinghai |
| GCA_049898915.1 | Pig | MSSA |  | t571 | China:Qinghai |
| GCA_049898885.1 | Pig | MSSA |  | t571 | China:Qinghai |
| GCA_049898865.1 | Pig | MSSA |  | t571 | China:Qinghai |
| GCA_049898825.1 | Pig | MSSA |  | t571 | China:Qinghai |
| GCA_049898745.1 | Pig | MSSA |  | t571 | China:Qinghai |
| GCA_049898725.1 | Pig | MSSA |  | t571 | China:Qinghai |
| GCA_049898645.1 | Pig | MSSA |  | t571 | China:Qinghai |
| GCA_049898625.1 | Pig | MSSA |  | t571 | China:Qinghai |
| GCA_049898605.1 | Pig | MSSA |  | t571 | China:Qinghai |
| GCA_049898585.1 | Pig | MSSA |  | t571 | China:Qinghai |
| GCA_049898505.1 | Pig | MSSA |  | t571 | China:Qinghai |
| GCA_049898485.1 | Pig | MSSA |  | t571 | China:Qinghai |
| GCA_049898465.1 | Pig | MSSA |  | t571 | China:Qinghai |
| GCA_049898385.1 | Pig | MSSA |  | t571 | China:Qinghai |
| GCA_049898365.1 | Pig | MSSA |  | t571 | China:Qinghai |
| GCA_049898345.1 | Pig | MSSA |  | t571 | China:Qinghai |
| GCF_024916525.1 | pig | MSSA |  | t571 | China:Guangdong |
| GCF_024916515.1 | pig | MSSA |  | t571 | China:Guangdong |
| GCF_053148535.1 | Pig | MRSA | XII | t571 | China:Guangdong |
| GCA_024589055.1 | Pig | MRSA | IX | t571 | China:Guangdong |
| GCA_024588975.1 | Pig | MRSA | IX | t571 | China:Guangdong |
| GCA_024588895.1 | Pig | MRSA | V | t571 | China:Guangdong |
| GCA_024588765.1 | Pig | MRSA | IX | t571 | China:Guangdong |
| GCA_024588575.1 | Pig | MRSA | IX | t571 | China:Guangdong |
| GCA_024588535.1 | Pig | MRSA | IX | t571 | China:Guangdong |
| GCA_024588215.1 | Pig | MRSA | XII | t571 | China:Guangdong |
| GCA_024588295.1 | Pig | MRSA | XII | t571 | China:Guangdong |
| GCF_053148595.1 | Pig | MRSA | XII | t571 | China:Fujian |
| GCA_002089115.2 | Human | MSSA |  | t571 | China:Guangdong |
| GCA_005931035.2 | Human | MSSA |  | t571 | China:Guangdong |
| GCA_023850215.1 | Human | MSSA |  | t571 | China:Hubei |
| GCA_023850045.1 | Human | MSSA |  | t571 | China:Hubei |
| GCA_028466185.1 | Human | MSSA |  | t571 | China:Shanghai |
| GCA_028466025.1 | Human | MSSA |  | t571 | China:Shanghai |
| GCA_028467165.1 | Human | MSSA |  | t571 | China:Shanghai |
| GCA_028467225.1 | Human | MSSA |  | t571 | China:Shanghai |
| GCA_028466585.1 | Human | MSSA |  | t571 | China:Shanghai |
| GCA_028467185.1 | Human | MSSA |  | t571 | China:Shanghai |
| GCA_028465955.1 | Human | MSSA |  | t571 | China:Shanghai |
| GCA_028584935.1 | Human | MSSA |  | t571 | China:Tianjin |
| GCA_028585545.1 | Human | MSSA |  | t571 | China:Tianjin |
| GCA_028466005.1 | Human | MRSA | V | t571 | China:Shanghai |
| GCA_026517685.1 | Human | MRSA | V | t571 | China:Jiangsu |
| GCA_024588515.1 | Human | MRSA | IX | t571 | China:Guangdong |
| GCF_002208555.1 | Human | MSSA |  | t571 | France |
| GCF_001696415.1 | Human | MSSA |  | t571 | France |
| GCF_002208535.1 | Human | MSSA |  | t571 | France |
| GCF_001696365.1 | Human | MSSA |  | t571 | France |
| GCA_028515905.1 | Goat | MSSA |  | t571 | France |
| GCA_028515785.1 | Horse | MSSA |  | t571 | France |
| GCA_028512655.1 | Dog | MSSA |  | t571 | France |
| GCA_028516125.1 | Bovine | MSSA |  | t571 | France |
| GCA_028516185.1 | Cat | MSSA |  | t571 | France |
| GCA_029766175.1 | Cow | MSSA |  | t011 | China:Jiangsu |
| GCA_029766165.1 | Cow | MRSA | V | t011 | China:Jiangsu |
| GCA_030220305.1 | Cow | MRSA | V | t011 | China:Jiangsu |
| GCA_030220345.1 | Human | MRSA | V | t034 | China:Jiangsu |
| GCF_010994155.1 | Cow | MRSA | V | t034 | China:Shanghai |
| GCF_010994215.1 | Cow | MRSA | V | t034 | China:Shanghai |
| GCA_030220365.1 | Human | MRSA | V | t034 | China:Shanghai |
| GCF_020881895.1 | Human | MRSA | V | t034 | China:Shanghai |
| GCA_028465975.1 | Human | HA_MRSA | V | t034 | China:Shanghai |
| GCA_028467325.1 | Human | HA_MRSA | V | t011 | China:Shanghai |
| GCA_028467025.1 | Human | HA_MRSA | IV | t034 | China:Shanghai |
| GCA_021479925.1 | Human | MSSA |  | t011 | China:Shanghai |
| GCF_003309525.1 | Human | MSSA |  | t034 | China:Shandong |
| GCF_003309445.1 | Pig | MSSA |  | t034 | China:Shandong |
| GCF_003309465.1 | Pig | MSSA |  | t011 | China:Shandong |
| GCA_028586035.1 | Human | MSSA |  | t011 | China:Tianjin |
| GCA_028584045.1 | Human | MSSA |  | t034 | China:Tianjin |
| GCA_028586585.1 | Human | MSSA |  | t034 | China:Tianjin |
| GCF_015767495.1 | Pig | MRSA | V | t034 | China:Beijing |
| GCA_030220325.1 | Human | MRSA | V | t034 | China:Beijing |
| GCF_053148575.1 | Pig | MRSA | V | t011 | China:Shanxi |
| GCF_053148555.1 | Pig | MRSA | V | t011 | China:Shanxi |
| GCF_049900345.1 | Pig | MRSA | V | t011 | China:Qinghai |
| GCF_049900105.1 | Pig | MRSA | V | t011 | China:Qinghai |
| GCF_002089055.1 | Human | MRSA | V | t034 | China:Guangdong |
| GCA_002089095.2 | Human | MSSA |  | t034 | China:Guangdong |
| GCA_008462245.1 | Human | MSSA |  | t034 | China:Guangdong |
| GCA_005931015.2 | Human | MSSA |  | t034 | China:Guangdong |
| GCA_005931055.2 | Human | MSSA |  | t034 | China:Guangdong |
| GCF_002407225.1 | Human | MRSA | V | t034 | China:Zhejiang |
| GCF_002307435.1 | Human | MRSA | V | t034 | China:Zhejiang |
| GCF_002723775.1 | Human | MRSA | V | t034 | China:Zhejiang |
| GCA_020702535.2 | Human | MSSA |  | t034 | China:Hubei |
| GCA_023848135.1 | Human | MSSA |  | t034 | China:Hubei |
| GCA_020702135.2 | Human | MSSA |  | t034 | China:Hubei |
| GCA_023855725.1 | Pig | MSSA |  | t034 | China:Hubei |
| GCA_025563015.1 | Pig | MSSA |  | t034 | China:Hubei |
| GCA_023855545.1 | Pig | MSSA |  | t034 | China:Hubei |
| GCA_023850745.1 | Human | HA-MRSA | V | t034 | China:Hubei |
| GCA_023850625.1 | Human | HA-MRSA | V | t034 | China:Hubei |
| GCA_020702575.2 | Human | HA-MRSA | V | t034 | China:Hubei |
| GCA_020702615.2 | Human | HA-MRSA | V | t034 | China:Hubei |
| GCA_023846315.1 | Human | HA-MRSA | V | t034 | China:Hubei |
| GCA_023855985.1 | pig | LA-MRSA | Unknown | t034 | China:Hubei |
| GCA_020702585.2 | pig | LA-MRSA | Unknown | t034 | China:Hubei |
| GCF_020702545.2 | Human | LA-MRSA | V | t034 | China:Hubei |
| GCA_026530815.1 | Human | MRSA | V | t034 | China:Hubei |
| GCA_026507155.1 | Human | MRSA | V | t034 | China:Hubei |
| GCA_026507215.1 | Human | MRSA | V | t034 | China:Hubei |
| GCA_026530815.1 | Human | MRSA | V | t034 | China:Hubei |
